# Analysis of maxillary teeth and soft tissue profiles among Tibetan and Han Chinese females with facial symmetry for orthodontic treatment planning

**DOI:** 10.3389/fsurg.2024.1384207

**Published:** 2024-07-31

**Authors:** Lijia Deng, Jinmei Lei, Minjie Li, Hongjie Song, Hong He

**Affiliations:** ^1^ Department of Stomatology, Chengdu Second People’s Hospital, Chengdu, China; ^2^ State Key Laboratory of Oral and Maxillofacial Reconstruction and Regeneration, Key Laboratory of Oral Biomedicine Ministry of Education, Hubei Key Laboratory of Stomatology, School & Hospital of Stomatology, Wuhan University, Wuhan, China; ^3^ Department of Orthodontics, School & Hospital of Stomatology, Wuhan University, Wuhan, China; ^4^ Department of Orthodontics, Stomatological Hospital, School of Stomatology, Southern Medical University, Guangzhou, China

**Keywords:** teeth, forehead, soft tissue, facial, orthodontic

## Abstract

The evaluation of maxillary teeth and soft tissue profiles is a critical component of orthodontic diagnosis and treatment planning. This study aimed to evaluate the correlation between the sagittal position of maxillary anterior teeth and facial profile esthetics among Tibetan and Han Chinese adult females for optimizing orthodontic diagnosis and treatment planning. A total of 100 Tibetan Chinese and 100 Han Chinese adult females with good facial symmetry were recruited. The smiling facial profile images with the maxillary central incisors and forehead in full view were taken based totally on the same standard. The photo measurement and head position were adjusted using the picture-enhancing software. The reference traces associated with forehead inclinations were utilized to assess the anteroposterior (AP) positions of the maxillary central incisors. The results showed that a round forehead was the dominant forehead shape for Tibetan (93%) and Han (55%) Chinese females. In Tibetan females, 85% of the maxillary central incisors were found to be located between the forehead's anterior limit line (the Gall line) and the goal anterior limit line (the Fall line), with 15% located posterior to the Fall line. This distribution manifested a strong association with incisor position and forehead inclination (R^2^* *= 0.742). In Han females, 83% of the maxillary central incisors were located between the Fall line and the Gall line, with 12% posterior to the Fall line and 5% anterior to the Gall line. The positions of the maxillary central incisors exhibited a strong relationship with forehead inclination (R^2^* *= 0.827). The maxillary central incisors were close to the aesthetic line in both ethnic groups, while forehead inclinations were correlated with AP maxillary incisor position. These findings demonstrated that there was a close relationship between the incisor position of Tibetan and Han females with facial symmetry and the forehead FFA factor, indicating a reference in oral hard and soft tissues for optimizing orthodontic diagnosis and treatment planning in terms of facial contour.

## 1 Introduction

The pursuit of facial beauty and harmonious appearance has become one of the primary orthodontic treatment goals (1). The evaluation of facial contour is divided into frontal and lateral components. The traditional cephalometric method can assess a patient's facial contour by quantifying the length and angle between bony landmarks. However, correcting the hard tissue in oral and maxillofacial regions at the ordinary cost does not always enhance the facial contour (2).

Many methods are currently used to measure and analyze facial contours by drawing bony landmarks on lateral cranial radiographs. Some studies have shown that solely measuring hard tissue is no longer sufficient to provide ideal information for the analysis and coordination of the beauty of the whole face, while the profiles of soft tissue cannot accurately replicate the state of affairs of teeth and bones. Consequently, high-quality analysis and treatment should be based on the combined measurement and analysis of both soft and hard tissues (3, 4). The soft and hard tissues measured by lateral cranial radiographs revealed that soft tissue modifications associated with tooth movement. The soft tissues masking difficult tissues had a high degree of variability as well as their own characteristics, so changes in hard tissues should no longer completely replicate the soft tissue profile. Another study reported a weak correlation between lateral cranial radiographs and profile photos (5). Therefore, orthodontists have started to pay special attention to the analysis of soft tissue profile, which is used as the primary tool for evaluating facial profile. Many researchers employ various dimension techniques to determine the average value of an everyday gentle tissue profile. Different races, nationalities, and age groups have unique, widespread values (6). In addition, there are many distinct dimension methods, and each holds its own advantages. Andrews et al. (7) proposed the six elements of orofacial harmony, which included a three-dimensional diagnostic and treatment planning. They conducted soft tissue analysis of the smiling profile of adult Caucasians with a good profile and analyzed the anteroposterior (AP) position of maxillary incisors using the forehead as a reference. They reported that adult white females’ foreheads with a good profile are an important indicator for locating maxillary incisors. Successively, equal research had been performed among Caucasian males (8). Gidaly et al. (9) investigated the optimal AP position of the maxillary central incisors and their relationship to the forehead in adult African American females. They discovered that the glabella vertical serves as a dependable anatomical landmark for identifying the AP maxillary incisal position in African American females. So far, there are no reports on other races. Researchers have argued that the relationship between the maxillary incisors’ AP position and lip lingual inclination is closely related to the coordinated profile. In contrast, the protrusion of the upper lip plays a significant role in the coordinated profile. Changes in the inclination and function of the maxillary incisors exert an immediate effect on the modifications of the upper lip (10–13).

There is no consistent concept of facial coordination, and standards of splendor vary greatly between countries and races (14). In China, a multi-ethnic country, all ethnic groups have specific facial structural characteristics because of their ethnic characteristics, geographical location, climate, and exceptional consuming habits. Tibetans, one of the minority nationalities in China, primarily live on plateaus with cold weather, low oxygen, low pressure, and strong ultraviolet radiation. Due to the harsh local weather and living conditions, their eating regimen is extremely simple and includes meals excessive in protein and calories, as well as coarse food. The incidence of malocclusion is extremely low, and the direct type is most common. Despite the differences and variations between Tibetan and Han Chinese adults, no updated studies have been reported regarding maxillary teeth and soft tissue profiles in these two ethnic groups.

In this study, Andrew's profile evaluation technique was used to measure and analyze the hard and soft tissue profile characteristics of Tibetan Chinese adult females with facial symmetry, which were then compared to Han Chinese adult females. The relationship between maxillary incisor position and forehead FFA point was explored to provide a guidance for optimizing orthodontic facial contour.

## 2 Materials and methods

### 2.1 Study object

This study involving human subjects was approved by the Clinical Research Ethics Committee of the School of Stomatology, Wuhan University (certificate 2022-B33). Based on facial profile photographs of students as they entered Sichuan Tibetan School, Sichuan Institute for Nationalities, and Wuhan University, a total of 100 Tibetan Chinese and 100 Han Chinese adult females, both with good facial symmetry, were recruited. Tibetan and Han females were assigned to the experimental and control groups, respectively. Inclusion standards were selected as below: aged 18–25 years old; lived in high altitude areas for a long time, the lineage of three generations was Tibetan for the experimental group; lived in low altitude areas for a long time, and the lineage of three generations was Han for the control group. There is no history of orthodontic treatment and cosmetic surgery. Three orthodontists with work experience over 5 years determined that these subjects had attractive profile appearances. Photographs of subjects with pleasing profiles were selected by these orthodontists based on calibration. If two out of the three judges reached a unanimous decision, the subjects were chosen. All individuals were aware that their images would be being used for this study.

### 2.2 Study method

All subjects were taken standardized lateral facial images. The digicam consisted of a tripod SLR camera (Nissan D700) and a matching flash device. The tripod was used to adjust the right peak in accordance with the height of the subject. To ensure a natural scale, a 70 mm ISO focal lens was selected. To keep away from the “red-eye effect” in the photo, the primary flash lamp was used to be constant on a tripod with a sidearm at a distance of 27 cm from the camshaft and 75 degrees from the upper right corner. Two auxiliary flashlights synchronized with the main flash were additionally used to illuminate the subjects’ faces and reduce unnecessary shadows. Another placing aspect was used as a secondary flash positioned at the back of the subject to illuminate the background and get rid of undesirable shadows on the face's contour. A natural head position was required when taking photos (15), and a mirror (20 cm × 35 cm) was vertically placed at 150 cm in front of the subject so that the challenge was once standing in front of the mirror, searching immediately into their own eyes, with naturally drooping arms. Subjects were required to fully expose the forehead, relax the lips, or smile while exposing the upper incisors.

### 2.3 Fixed-point measurement

**Figure 1:**
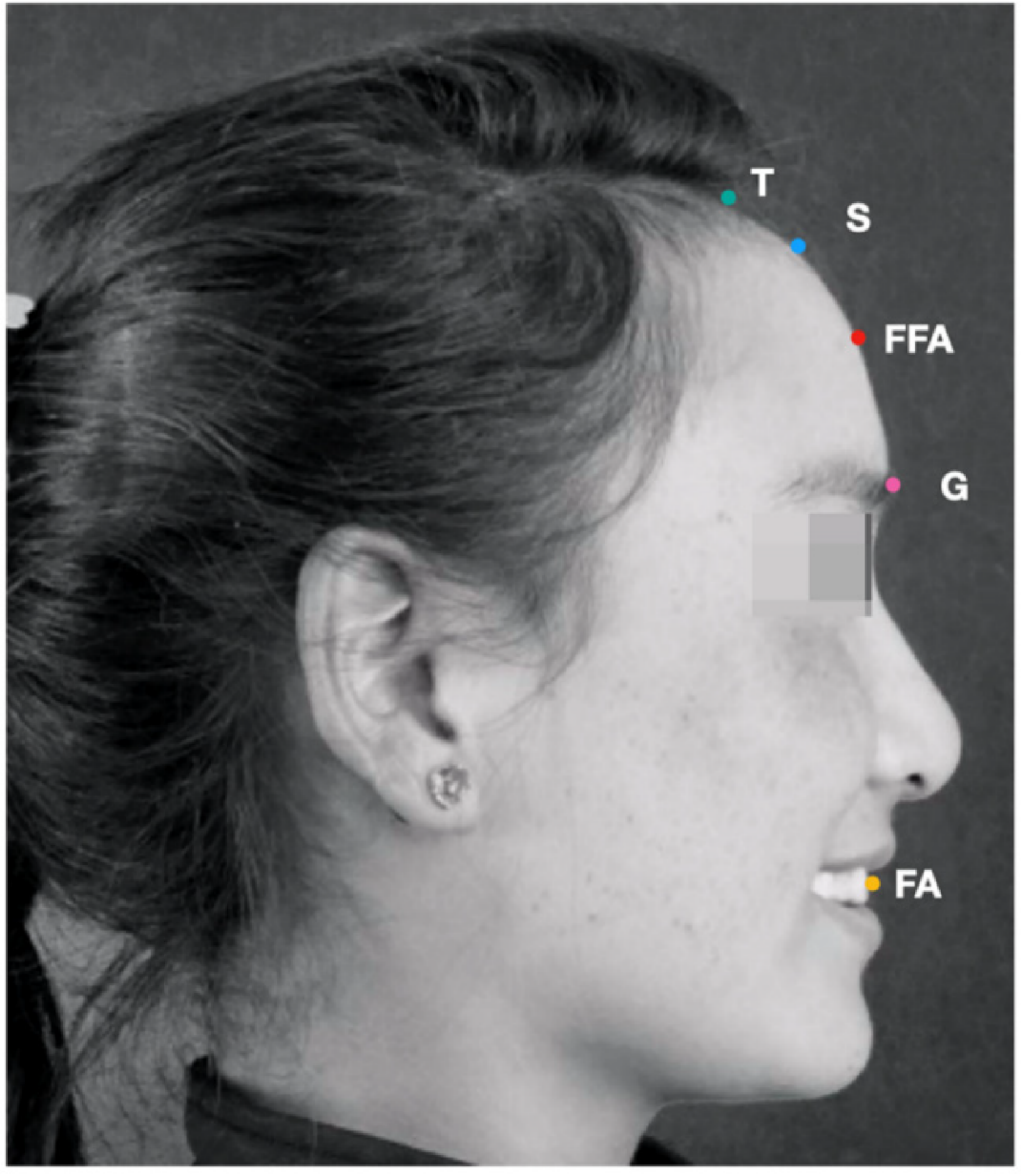
In accordance with the landmarks determined by andrew's profile analysis method, each subject was marked on the right side of the photo. The forehead landmarks include the trichion point (T point), superion point (forehead most protruding point, S point), glabella point (G point), forehead facial-axis point (FFA point), and facial-axis point of maxillary central incisors (FA point).

**Figure 2:**
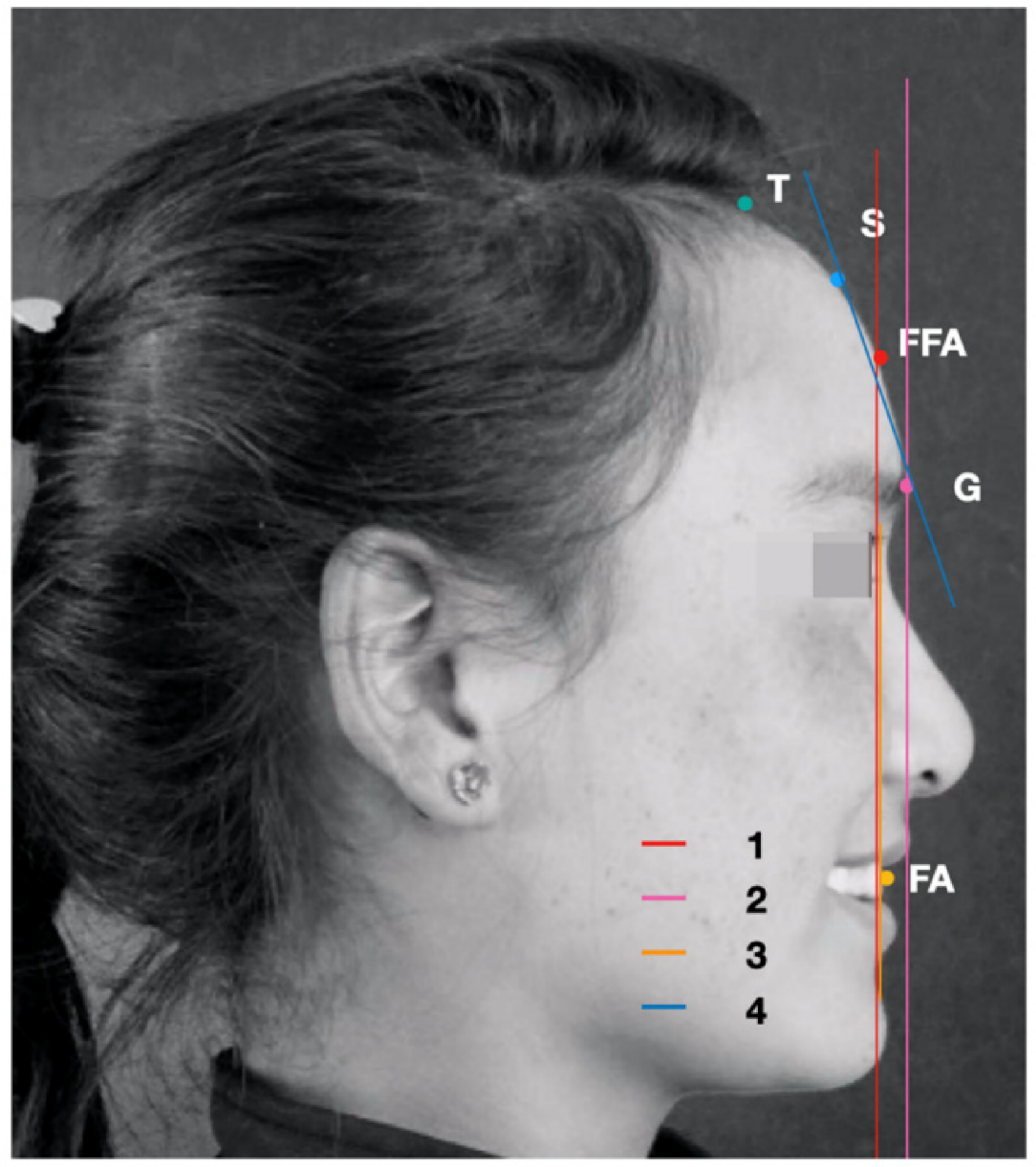
All the landmarks were marked on the median sagittal plane of the head, and four straight lines were drawn, respectively. Lines 1 and 2 were the vertical lines passing through the FFA point and G point, respectively, i.e., the plane of the Fall and Gall lines. Line 3 is the vertical line passing through the FA point. The line connected between the S point and G point was line 4. When the upper incisor (line 3) located before the FFA point (line 1), it was positive; otherwise, it was negative.

According to the landmarks determined in Andrew's profile analysis method, each subject was used to be marked in Figure 1 and Table 1. The forehead landmarks included the trichion point (T point), superion point (forehead most protruding point, S point), glabella point (G point), forehead facial-axis point (FFA point), and facial-axis point of maxillary central incisors (FA point). The T point is the midpoint of the hairline. When the modern brow contour point is flat, the T point is the S point. The point between the eyebrows is the lowest part of the forehead. When the forehead is round or angular, the S point is the most prominent point. The FFA point is the midpoint of the forehead. When the frontal contour is flat, the FFA point is used to be positioned at the center of the line between the hairline and the eyebrow. The FFA point is located at the midpoint of the line between the S point and the brow. The incisor landmark is positioned at the central point of the clinical crown. All the landmarks were marked on the median sagittal plane of the head, and four straight lines were drawn, respectively (Figure 2 and Table 1). Lines 1 and 2 are the vertical lines passing through the FFA point and G point, respectively, i.e., the plane of the Fall and Gall lines. Line 3 is the vertical line passing through the FA point. When the upper incisor (line 3) is once placed in front of the FFA point (line 1), it is positive; otherwise, it is negative. The line connecting the G and S points is line 4. The angle between line 1 and line 4 is measured with a protractor to define the inclination of the forehead (accurate to 0.5 degrees).

**Table 1:** Abbreviations for the anatomical landmarks identified by andrews’ profile analysis method.

The anatomical marker	Abbreviations
The forehead landmarks included the trichion point	T point
The forehead most protruding point	S point
The glabella point	G point
The Forehead Facial Axis point	FFA point
The facial-axis point of maxillary central incisors	FA point
The anteroposterior position	AP position
The goal anterior limit line (The vertical lines passing through the FFA point)	Fall line
The forehead's anterior limit (The perpendicular passing through the G-point, parallel to FALL)	Gall line

### 2.4 Statistical analysis

The mean and standard deviation (SD) of the AP position of the maxillary central incisor relative to the forehead and the frontal inclination perspective were calculated by SPSS 20.0 statistical software. A *p*-value < 0.05 indicated a statistically significant difference. Simple linear regression was used to analyze the correlation between FA and frontal inclination of maxillary central incisors. The confidence interval was set at 95%. Consistency test: 20 participants were randomly selected from the measurement data of the two groups. The same experimenter repeated the fixed point and measurement twice every 3 weeks, and the intra-group correlation coefficient (ICC) was used for calculation.

## 3 Results

In this study, the consistency showed that the measurement system error was very small, and the two measurement results’ consistency was excessive (Table 2). In the Tibetan female group, 93% had round foreheads, while it was angular in 7%, with an inclination of 5.5–25 degrees and an average of 14.9 ± 3.27 degrees. In the Han female group, 55% had round foreheads, while 37% were angular and 8% were straight, with an inclination of 8 to 27 degrees and an average of 16.6 ± 4.3 degrees. There was a significant variation between the two groups (*p* = 0.012) (Table 3).

**Table 2:** The consistency of the intragroup correlation coefficient (ICC).

*N* = 20	Intraclass correlation	95% Confidence interval	
		Lower bound	Upper bound
Inclination	0.994	0.985	0.998
Distance	0.999	0.997	0.999

Twenty participants were randomly selected from the measurement data of the experimental and control groups, respectively. The same experimenter repeated the fixed-point and measurement twice every three weeks.

**Table 3:** Comparison of incidence rate of forehead shape between the experimental and control groups (*N* = 100).

	Experimental group (Tibetan)	Control group (Han)
Round forehead (%)	93	55
Angular forehead (%)	7	37
Straight forehead (%)	0	8
Forehead inclination (degree)	14.9 ± 3.27	16.6 ± 4.3
*p* value	0.012	

Using the forehead as the reference, 85% of the maxillary central incisors were found to be located between the Fall line and the Gall line plane in the experimental group, and 15% were located posterior to the Fall line. The distance between the maxillary central incisors’ AP position and the Fall line plane ranged from −4.4 to 4.8 mm. In the control group, 83% of the maxillary central incisors were situated between the Fall line and the Gall line, whereas 12% were situated posterior to the Fall line and 5% were situated anterior to the Gall line. The distance between the AP position of the maxillary central incisors and the fall line plane ranged from −3.4 mm to 11.4 mm, with a significant difference between the two groups (*p* = 0.00) (Table 4).

**Table 4:** Analysis of the anteroposterior (AP) position of maxillary central incisors with the forehead as a reference (*N* = 100).

Position of maxillary central incisor	Experimental group (Tibetan)	Control group (Han)
Between the fall and gall line (%)	85	83
Posterior to the fall line (%)	15	12
Anterior to the gall line (%)	0	5
*p* value	0.000	

**Figure 3:**
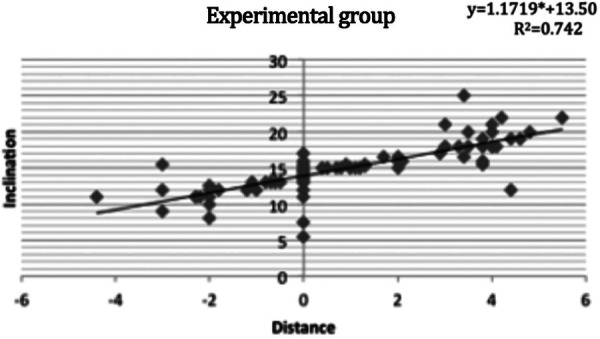
The correlation between the FFA of maxillary central incisors and frontal inclination in the experimental group using simple linear regression analysis and its linear regression equation.

**Figure 4:**
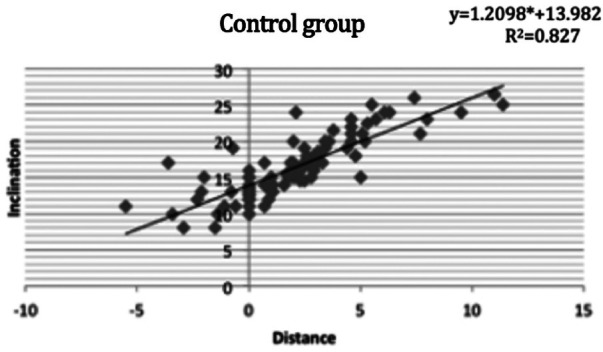
The correlation between the FFA of maxillary central incisors and frontal inclination in the control group using simple linear regression analysis and its linear regression equation.

The position of the maxillary central incisor and the frontal inclination was correlated between the experimental (R^2^ = 0.742, Figure 3) and control (R^2^ = 0.827, Figure 4) groups. The correlation between the FFA of maxillary central incisors and frontal inclination was analyzed by simple linear regression. When the frontal inclination of the experimental group was 13.5 degrees, the FA point of the maxillary central incisors overlapped on the Fall line plane, and the FA point of the maxillary incisors moved forward 0.85 mm for every 1-degree increase of the inclination. In the control group, when the frontal inclination was about 14 degrees, the FA point of maxillary central incisors overlapped with the Fall line plane, and the FA point of maxillary incisors moved forward 0.74 mm for each additional degree.

## 4 Discussion

A harmonious face should include the forehead, as well as the jaw shape, lip position, and nose. To create a balanced appearance, each element is considered to be in harmony. Moreover, the contour of the forehead must be considered as part of the overall facial contour (1). Although cephalometric analyses have mainly addressed structures below the forehead, this region should be recognized and included in a thorough assessment. The relationship between the sagittal position of the upper incisors and facial profile esthetics has previously been suggested (16): When creating treatment plans for patients with orthodontic issues, determining the proper maxillary incisor position has become crucial. For sole hard tissue measurements, soft tissue alterations are not always consistent with changes in hard tissue, making it difficult to predict changes in soft tissue using only hard tissue analyses. AP position of maxillary incisors with reference to the forehead was analyzed in this study along with a profile-conducted soft and hard tissue analysis of the smiling profile, and Andrew's profile evaluation approach was used to compare the profile of Tibetan and Han Chinese adult females with facial symmetry. Young females aged 18–25 years old were chosen to prevent skeletal growth from affecting the study's findings. Certain differences in forehead shape and inclination between Tibetan and Han females were confirmed. Most of the maxillary central incisors in the two groups were placed between the Fall line plane and Gall line plane, which was in line with Andrews’ theory. Accordingly, the Fall lime plane can be considered as a profile aesthetic line. However, due to differences in brow structure and protrusion between the two groups, there was a sizable distinction in the FA point of maxillary central incisors.

Studies (7, 17) suggest that the position of the forehead is crucial for a coordinated profile and can be used to diagnose or predict the most fulfilling function of the maxillary central incisors. The forehead can be divided into three shapes: round, straight, and angular. Most of the subjects in the two groups had FA points between the Fall line plane and the Gall line plane, which supported Andrew's concept that the former forehead represented the aesthetic reference line and was also applicable to Tibetan and Han ethnic groups. When the forehead was 13.5 degrees in the Tibetan group, the FA point of the maxillary central incisor overlapped with the Fall line plane, whereas the Han group's was 14 degrees. The two sets of data showed no statistically significant differences. Both Tibetan and Han Chinese females had smaller forehead angles than Caucasian measurements (7), as did African American females (9). In the experimental and control groups, except for 5% of the maxillary central incisors in the Han group that were located before the Gall line plane, the rest of the maxillary central incisors were behind the Fall line plane, indicating that the majority of females with facial symmetry had a straight face type. In addition, all participants in the experimental group had a straight face type, which conforms to a previous study on the head and face characteristics of Tibetan females (18). The subjects’ upper and lower lips are located at the back of the tangent line, passing through the most protruding point of the nose and chin, also known as Ricketts aesthetic line. These findings suggested that most Tibetan young adults have a straight face and thus meet the aesthetic requirements. It should be noted that our findings contradict those of African American females (9), who found a greater anterior forehead inclination among this population, implying that the optimal location for the FA point is ahead of the Gall line.

Andrew's six elements are the standard for perfect tooth and maxillofacial coordination, as well as the goal of orthodontic treatment. This approach can predict the movement of teeth and jaw and changes in soft tissue in the process of orthodontic treatment. Therefore, this study is promising in providing a better understanding of the high-quality role of their teeth or jaw, which assists in clinical diagnosis and the determination of optimal orthodontic treatment.

## 5 Conclusion

The conclusions include: (1) the inclination of the forehead varied significantly between Tibetan and Han ethnic groups, with Tibetans having rounded foreheads; (2) the maxillary central incisors in Tibetan and Han Chinese females with facial symmetry positioned close to the Fall line plane of Andrews profile analysis; (3) there was a correlation between frontal inclination and AP position of maxillary central incisors, and the forehead is an important mark in profile evaluation; and (4) most of the maxillary central incisors in the two ethnic groups positioned between the Fall line plane and the Gall line plane, and the Fall line plane can be considered as a profile aesthetic line.

## Data Availability

The original contributions presented in the study are included in the article/Supplementary Material, further inquiries can be directed to the corresponding author.
